# Automated Classification of Mild Cognitive Impairment by Machine Learning With Hippocampus-Related White Matter Network

**DOI:** 10.3389/fnagi.2022.866230

**Published:** 2022-06-14

**Authors:** Yu Zhou, Xiaopeng Si, Yi-Ping Chao, Yuanyuan Chen, Ching-Po Lin, Sicheng Li, Xingjian Zhang, Yulin Sun, Dong Ming, Qiang Li

**Affiliations:** ^1^School of Microelectronics, Tianjin University, Tianjin, China; ^2^Academy of Medical Engineering and Translational Medicine, Tianjin University, Tianjin, China; ^3^Tianjin Key Laboratory of Brain Science and Neural Engineering, Tianjin University, Tianjin, China; ^4^Institute of Applied Psychology, Tianjin University, Tianjin, China; ^5^Graduate Institute of Biomedical Engineering, Chang Gung University, Taoyuan, Taiwan; ^6^Department of Computer Science and Information Engineering, Chang Gung University, Taoyuan, Taiwan; ^7^Institute of Neuroscience, National Yang Ming Chiao Tung University, Hsinchu, Taiwan

**Keywords:** mild cognitive impairment, white matter connectivity, Alzheimer’s disease, early diagnosis, feature extraction, machine learning

## Abstract

**Background:**

Detection of mild cognitive impairment (MCI) is essential to screen high risk of Alzheimer’s disease (AD). However, subtle changes during MCI make it challenging to classify in machine learning. The previous pathological analysis pointed out that the hippocampus is the critical hub for the white matter (WM) network of MCI. Damage to the white matter pathways around the hippocampus is the main cause of memory decline in MCI. Therefore, it is vital to biologically extract features from the WM network driven by hippocampus-related regions to improve classification performance.

**Methods:**

Our study proposes a method for feature extraction of the whole-brain WM network. First, 42 MCI and 54 normal control (NC) subjects were recruited using diffusion tensor imaging (DTI), resting-state functional magnetic resonance imaging (rs-fMRI), and T1-weighted (T1w) imaging. Second, mean diffusivity (MD) and fractional anisotropy (FA) were calculated from DTI, and the whole-brain WM networks were obtained. Third, regions of interest (ROIs) with significant functional connectivity to the hippocampus were selected for feature extraction, and the hippocampus (HIP)-related WM networks were obtained. Furthermore, the rank sum test with Bonferroni correction was used to retain significantly different connectivity between MCI and NC, and significant HIP-related WM networks were obtained. Finally, the classification performances of these three WM networks were compared to select the optimal feature and classifier.

**Results:**

(1) For the features, the whole-brain WM network, HIP-related WM network, and significant HIP-related WM network are significantly improved in turn. Also, the accuracy of MD networks as features is better than FA. (2) For the classification algorithm, the support vector machine (SVM) classifier with radial basis function, taking the significant HIP-related WM network in MD as a feature, has the optimal classification performance (accuracy = 89.4%, AUC = 0.954). (3) For the pathologic mechanism, the hippocampus and thalamus are crucial hubs of the WM network for MCI.

**Conclusion:**

Feature extraction from the WM network driven by hippocampus-related regions provides an effective method for the early diagnosis of AD.

## Introduction

Alzheimer’s disease (AD) is a chronic neurodegenerative disease with irreversible progression (Sperling et al., 2011; Hyman et al., 2012). Mild cognitive impairment (MCI) is the prodromal stage of AD (Petersen et al., 1999; Gauthier et al., 2006). The primary clinical manifestation of MCI is memory loss (Petersen et al., 2001). Since the progression of AD is irreversible and the treatment of AD has little effect, the detection of MCI is expected to find out the high risk of AD and further prevent its occurrence (Jacobs et al., 2013; Wang et al., 2013). However, the subtle changes of brain microstructure in MCI make it challenging to distinguish the disease from conventional radiography (Pellegrini et al., 2018). Therefore, establishing reliable biomarkers to diagnose MCI in the early stages remains challenging.

According to the biomarker modeling of AD, white matter demyelination has been proved to appear earlier during AD progression than abnormal changes of gray matter and functional connectivity (Zhuang et al., 2012; Lee et al., 2015). Recent studies have shown that genes and protein molecules ultimately cause microstructure changes in specific white matter fibers (Yu et al., 2021; Zhao et al., 2021). Thus, white matter degeneration is a valid biomarker for MCI. Diffusion tensor imaging (DTI) could detect subtle structural changes in white matter fibers, facilitating large-scale non-invasive screening (Tournier, 2019). The DTI index, mean diffusivity (MD), and fractional anisotropy (FA) describe fiber tracts’ state.

Machine learning offers a systematic approach to developing advanced, automatic, and objective classification frameworks for MCI diagnosis. The classification framework mainly includes feature extraction and classification algorithms (Rathore et al., 2017). Although there are plenty of studies on AD classification, there is insufficient research on MCI (Shatte et al., 2019). The classification accuracies for AD were all around 80%, while the accuracies for MCI were only about 60% (Wee et al., 2011; Dyrba et al., 2013, 2015a; Nir et al., 2015; Prasad et al., 2015; Dou et al., 2020). The main reason is that whole-brain changes in AD are so significant that can be classified with high accuracy by the features of white matter, gray matter, and functional connectivity. In contrast, changes in MCI are not obvious for the whole brain. However, the progression of AD is irreversible. Early diagnosis of MCI fascinates high-risk screening of AD in time.

Previous studies focused on the classification algorithm to improve classification performance. Existing researchers found that k-nearest neighbor (KNN) (Ebadi et al., 2017), random forest (RF) (Maggipinto et al., 2017; Wang et al., 2018), and support vector machine (SVM) (Cui et al., 2012; Demirhan et al., 2015; Dyrba et al., 2015a; Nir et al., 2015; Xie et al., 2015; Ahmed et al., 2017) have achieved better classification performance for MCI when taking the white matter as a feature. KNN is based on Euclid’s theorem and is classified by measuring the distance between different features. RF integrates many decision trees into a forest and combines them for predicting. SVM is based on statistical theory to solve two classification problems, mainly introducing kernel function to solve the problem of linear inseparability. However, previous studies only attached importance to algorithms while neglecting the features. In fact, selecting the appropriate modality in the data and extracting suitable features are usually more important than the underlying algorithm (Zhang et al., 2021).

The white matter feature extraction methods for MCI classification mainly include specific fiber tracking and the whole-brain white matter network (WM network). The specific fiber tracking method takes features using the fiber tracts that converge between localized brain regions and calculates the DTI index of voxels on a single fiber tract. However, this method focused on a specific fiber tract and had low classification accuracy (Nir et al., 2015; Dou et al., 2020). In the whole-brain white matter connectivity network approach, DTI images were segmented into several anatomical regions and features based on the metrics calculated from the fibers within these regions (Wee et al., 2011; Prasad et al., 2015). Recent studies had started to use multimodality for feature extraction. DTI, T1-weighted (T1w), and resting-state functional MRI (rs-fMRI) images from different modes were used to capture information from different perspectives (Hinrichs et al., 2011; Liu et al., 2014). However, most studies had concatenated the features from different modalities. The disadvantage is that all features are treated equally, and it provides no way to account for the diverse nature of features extracted from different modalities (Zhang et al., 2011, 2012; Dyrba et al., 2015b). Therefore, the urgent problem for MCI classification is to combine with different modalities before training and develop a dedicated feature fusion strategy.

To address this issue, the brain network theory was introduced. First, the brain can be seen as a network of spatially dispersed brain areas that share information continuously in functional connectivity (Sporns and Betzel, 2016). The different brain regions are connected and have synchronization within the same activation pattern. Different brain regions are temporally synchronized under the same activation pattern by the white matter pathway (Gu et al., 2015). Second, the primary clinical manifestation of MCI is memory loss (Rugg and Vilberg, 2013). The hippocampus is the crucial area for memory processing. Therefore, investigating the abnormal white matter network related to the hippocampus could promote the detection of MCI in time (Benoit and Schacter, 2015). Furthermore, previous studies have also demonstrated that white matter degeneration leads to abnormalities in the functional connectivity of the corresponding brain regions (Kleinschmidt and Vuilleumier, 2013). In the default mode network, damage to the white matter fibers such as fornix and cingulum could weaken the functional connectivity of the medial temporal lobe and precuneus to the hippocampus (Buckner and DiNicola, 2019). Therefore, we hypothesized that white matter connectivity between brain regions associated with the hippocampus could be an important biomarker for MCI recognition. Features extraction of white matter network through hippocampus-related regions would help to improve the MCI classification performance.

This study aimed to extract the effective features of white matter connectivity networks driven by regions related to the hippocampus and improve classification performance. In our study, the elderly people were divided into MCI group and normal control (NC) group. First, the DTI data were obtained, and the orientation distribution function (ODF) was calculated by constrained spherical deconvolution (CSD). Based on the ODF, the fiber tracts of the whole brain were constructed. Second, the DTI index of whole-brain white matter connectivity between each brain region under the automated anatomical labeling (AAL) was calculated to construct the MD and FA brain structure networks as features. Furthermore, the hippocampus was used as the seed point to define the regions of interest (ROIs). Brain regions with high correlation to the hippocampus in functional connectivity were obtained as ROIs compared with the AAL template. Finally, whole-brain white matter connectivity networks were screened by ROIs related to the hippocampus. Different classifiers (e.g., SVM, KNN, and RF) were used to validate the classification performance of the extracted features. The recursive feature elimination (RFE) ranked these features’ contribution to analyze the pathological mechanisms of MCI. Our study proposed a method of feature extraction to improve the MCI classification performance. The study also revealed the pathological mechanisms of MCI by the ranked contribution of features. It would provide effective early aid to MCI diagnosis.

## Materials and Methods

### Participants

After excluding the left-handed and other brain injury history, a total of 96 subjects met the criteria for inclusion (48 male and 48 female; age: 80.6 ± 5.4 years, mean ± std). All participants were provided written informed consent based on the Helsinki Declaration. The experimental protocol was approved by the Institutional Review Board of Tianjin University and the Ethics Committee of Chang Gung University.

### Neuropsychological Behavior Testing

All subjects were tested on the clinical dementia rating (CDR) and the mini-mental status examination (MMSE) scale. The entry criteria for MCI diagnosis were as follows: (i) CDR = 0.5; (ii) 24 ≤ MMSE < 30 for well-educated subjects (education years ≥ 6) or 19 ≤ MMSE < 24 for less-educated subjects (education years < 6). The MCI group was 81.3 ± 3.6 (mean ± std) years with education duration of 5.9 ± 5.4 years (mean ± std). There was no difference between groups in age and education duration (Table 1).

**TABLE 1:** Demographic and neuropsychological information of the MCI and NC groups.

	NC (*n* = 54)	MCI (*n* = 42)	*p*-value
Gender (m/f)	27/27	21/21	1.0000^a^
Age (year)	80.0 ± 6.3^b^	81.3 ± 3.6^b^	0.6353^c^
Education (year)	6.5 ± 4.1^b^	5.9 ± 5.4^b^	0.1530^c^
CDR	0	0.5	<0.0001^c^
MMSE	28.0 ± 1.8^b^	24.9 ± 2.8^b^	<0.0001^c^

*^a^Chi-square test. ^b^Mean ± std. ^c^Rank sum test.*

### Magnetic Resonance Imaging Data Acquisition

The DTI, T1w, and rs-fMRI images of each subject were acquired without personal information. The DTI with 30 diffusion encoding directions was acquired using echo planar imaging (EPI) sequence with parameters: *b*-value = 1,000 s/mm^2^, TR/TE = 11,000/104 ms, field of view = 192 × 192 mm^2^, matrix size = 128 × 128, slice thickness = 2 mm, voxel size = 2 × 2 × 2 mm^3^, number of slices = 70, and number of excitations = 1. The rs-fMRI was acquired using gradient EPI sequence with parameters: TR/TE = 2,500/27 ms, flip angle = 77°, band width = 2,400 Hz/pixel, field of view = 260 × 260 mm^2^, matrix size = 64 × 64, voxel size = 3.4 × 3.4 × 3.4 mm^3^, number of slices = 43, scan time = 360 s, and time points = 180. The T1w imaging was acquired using a 3D magnetization prepared rapid gradient EPI sequence with parameters: TR/TE = 2,530/3.5 ms; TI = 1,100 ms; field of view = 260 × 260 mm^2^; matrix size = 256 × 256; slice thickness = 1 mm; voxel size = 1 × 1 × 1 mm^3^; and number of slices = 192.

### White Matter Structure Network Establishment and Feature Extraction

**FIGURE 1:**
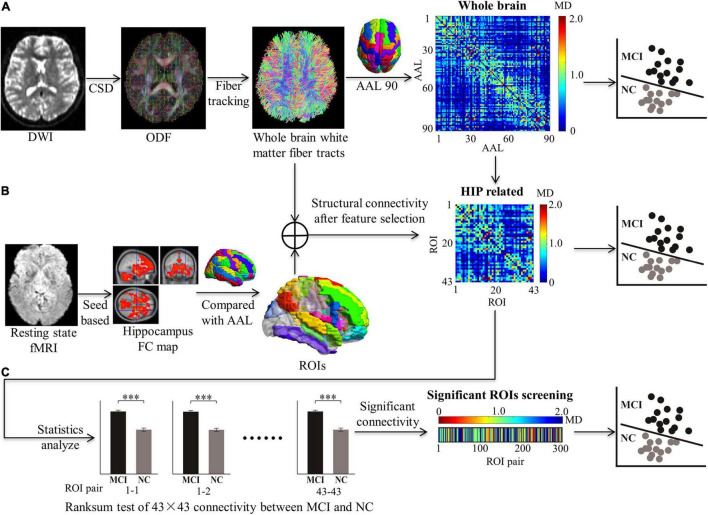
Feature extraction in structural connectivity map driven by hippocampus related ROIs. Three kinds of features were extracted for MCI classification, including: **(A)** The whole brain WM network was acquired from diffusion MRI with all 90 × 90 AAL regions. **(B)** The HIP related WM network was selected by 43 × 43 ROIs from the whole brain WM network. **(C)** The significant HIP related WM network was acquired (*p < 0.05, **p < 0.005, and ***p < 0.0005; ranksum test with bonferroni correction).

The feature extraction of the white matter structure network for MCI classification consists of the following steps (Figure 1):

1. MRI data preprocessing.
2. Reconstruction for white matter fiber tracts by orientation distribution function (ODF). Based on DTI, the ODF was calculated by CSD.
3. Establishment for whole-brain structure network. DTI indexes (MD and FA) of white matter connectivity between the AAL 90 brain region were calculated to establish the whole-brain structure network as the full feature for MCI classification.
4. Feature selection driven by hippocampus-related ROIs. Brain regions with significant functional connectivity to the hippocampus in rs-fMRI were selected as ROIs. The ROIs are used to screen features for the white matter structural network.
5. Machine learning for MCI recognition. Different classifiers (e.g., SVM, KNN, and RF) were used to test the features for MCI recognition.
6. Performance comparison for searching optimal features and classifiers. Classification performances were validated to prove that our feature extraction method was effective. Pathological mechanisms were analyzed with feature sort.

#### Magnetic Resonance Imaging Data Preprocessing

The DWI data were denoised and corrected for Gibb’s ringing using MRtrix3^1^ and then motion-corrected, and the eddy current distortion was corrected using the eddy tool in FSL (v5.0.11).^2^ Next, a brain mask was constructed using the Brain Extraction Tool in FSL, and the diffusion tensor fitting was performed at each voxel within the brain mask to generate DTI index maps. The rs-fMRI data were temporal band-pass filtered (0.01–0.10 Hz) and detrended (both linear and quadratic trends). 3D geometrical displacement was used to correct for head motion. Spatial smoothing was performed with a Gaussian filter kernel (FWHM = 6 mm). The entire process was performed using the Statistical Parametric Mapping (SPM) software package,^3^ in which the head motion parameters, global signals, white matter signals, and cerebrospinal fluid signals were obtained and combined into a complete covariate. Covariate from functional signals was then removed using the Resting-State fMRI Data Analysis Toolkit (REST).^4^

#### Reconstruction for White Matter Fiber Tracts

To reconstruct the whole-brain white matter fiber tracts of each subject, fibers were tracked in DTI data (Supplementary Figure 1). (1) The ODF necessary for the fiber tracking algorithm can be obtained using the MRtrix software (see text footnote 1). The CSD was used with maximum spherical harmonic degree = 6 during this process. (2) The ODF was integrated into DSI Studio^5^ to achieve a 3D reconstruction of the whole brain’s white matter fiber connectivity. The tracking parameters were as follows: Number of tracts = 100,000, Qa_threshold = 0.04, max angle = 60, length constraint = 20.0–450.0 mm, step size = 1.00 mm, and smoothness = 0.5 mm. Seed direction was set to random, and seed position was subvoxel.

#### Establishment for Whole-Brain Structure Network

Since the AAL is located in the MNI152 standard space, the transfer matrixes between the standard space and native space (Supplementary Figure 2) were required to obtain the AAL brain region for each subject space. (1) The b0 image from the DTI image served as the native space. The T1w image after skull stripping in FSL served as the structure space. The MNI152 template from FSL served as the standard space. (2) With the high-resolution T1w image as the reference, FMRIB’s Linear Image Registration Tool (FLIRT) was used to obtain the transition matrix from native space to structural space (T_NS_) and the transition matrix from standard space to structural space (T_MS_). The transition matrix from native space to standard space was T_NM_ = T_NS_ × T_MS_^–1^. (3) The transition matrix from standard space to native space was T_MN_ = T_MS_ × T_NS_^–1^.

To establish the whole-brain WM network, a 90 × 90 matrix for the whole brain divided by the AAL template was calculated (Supplementary Figure 1). (1) The AAL 90 brain regions in the MNI152 standard space were transformed to each subject’s space separately by applying transfer matrix T_MN_. (2) For the 90 × 90 matrix, every element records the mean DTI index (MD or FA) of fiber tracts between every two of the AAL 90 brain regions. Also, the whole-brain WM networks were established, including the MD connectivity 90 × 90 matrix and the FA connectivity 90 × 90 matrix. (3) Whole-brain WM networks of 54 NC and 42 MCI were established separately as entire features for MCI recognition.

#### Feature Selection Driven by Hippocampus-Related Regions of Interest

The primary clinical symptom of MCI is memory loss, and the hippocampus is closely associated with memory. So, the hippocampus was chosen as a seed point to calculate the brain regions that had significant functional connectivity (Supplementary Figure 3). The concrete steps were as follows: (1) All participants’ rs-fMRI images were transferred from the native space to the standard space. (2) The left hippocampus (30, −16, −14) and right hippocampus (−30, −16, −14) with a radius of 3 mm served as the seed points to create the FC map of each participant in the MNI152 standard space. With the same time series, brain regions that activated correlation with hippocampus were calculated in software REST. The Fisher Z transformation was applied so that the results of each participant followed normal distributions. (3) After individual-level analysis, group analysis for MCI and NC was conducted separately by the second-level analysis in SPM. A one-sample *T*-test and a familywise error correction were employed to revise the statistics of the group analysis results. (4) Significant area (*p* < 0.05) with the threshold (*T*-value > 10, size > 27) was selected to obtain the brain regions associated with the functional connectivity of the hippocampus at the group level.

To confirm the locations of the hippocampus-related ROIs, the following steps were conducted: (1) The functional connectivity maps of the MCI and NC groups were compared with AAL templates. The percentage of overlapping voxels taken in the AAL was calculated (Supplementary Figure 3). (2) To select as many ROIs as possible in the AAL and prevent false positives, a volume percentage threshold of 10% was set. If one of the 90 regions in the AAL had a volume fraction greater than the threshold value, the brain region in the AAL was selected as an ROI (Supplementary Figure 4). The selected ROIs are listed in Supplementary Table 1, including their abbreviations. (3) Totally 43 ROIs related to the hippocampus were used to extract features of the whole-brain WM network (90 × 90 matrices), and the HIP-related WM network (43 × 43 matrices) was established. The rank sum test with Bonferroni correction of HIP-related WM network between MCI and NC was used to extract significant features. The significant HIP-related WM network was acquired (300 vectors) (Figure 1).

#### Machine Learning for Mild Cognitive Impairment Recognition

**FIGURE 2:**
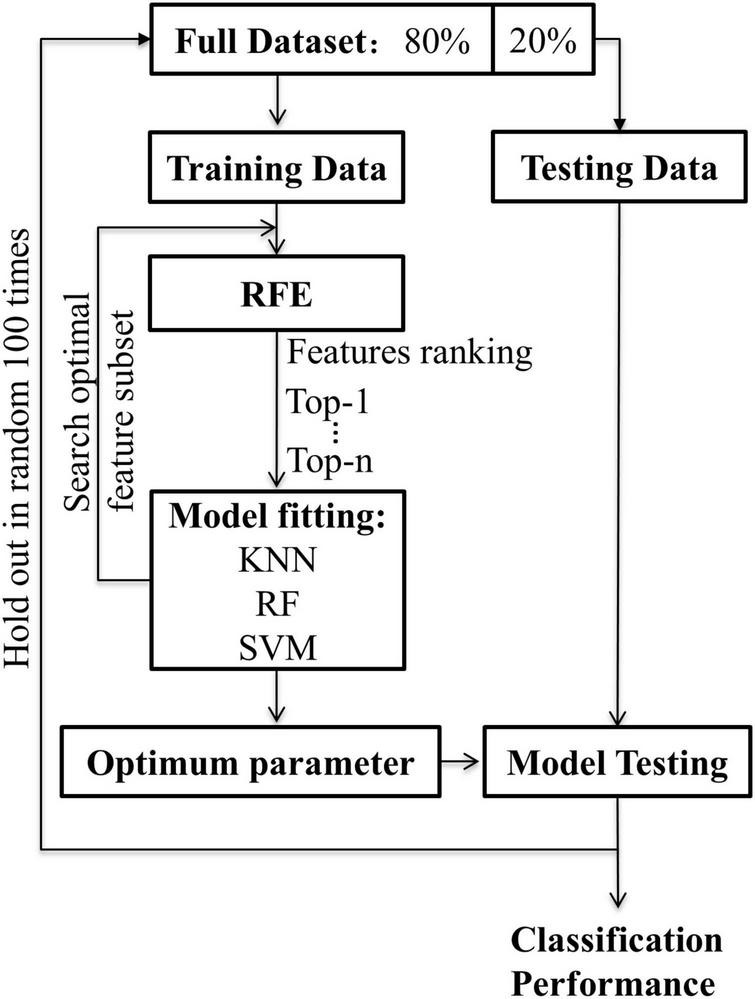
The flowchart for machine learning. Recursive feature elimination was used to search for the optimal feature subset. The classification algorithm was respectively used KNN, RF, SVM (linear, poly, rbf, sigmoid). The hold-out method was repeated 100 times randomly with 80% of the data for training and 20% for testing.

To train features of different WM networks for MCI classification, the process of machine learning was as follows (Figure 2): (1) The whole-brain WM network, the hippocampus-related WM network (HIP-related WM network), and the significant HIP-related WM network were separately used as features for machine learning. The recursive feature elimination (RFE) algorithm was used for both dimension reduction and features ranking. (2) The machine learning methods included SVM, KNN, and RF. For SVM, four different kernel functions were tried, namely, linear kernel (linear), polynomial kernel (polynomial), radial basis function kernel (rbf), and sigmoid kernel (sigmoid). For KNN, neighbors of 1, 3, 5, and 7 were used. For RF, 50, 100, 150, and 200 decision trees were used. (3) The hold-out method was used to evaluate classification performance; 80% of the data set was selected as the training set and the remaining 20% as the test set. This process was repeated 100 times randomly to obtain the average classification accuracy and the area under the curve (AUC). All the above experiments were based on the Scikit-Learn library.^6^

#### Performance Comparison for Searching Optimal Feature and Classifier

**FIGURE 3:**
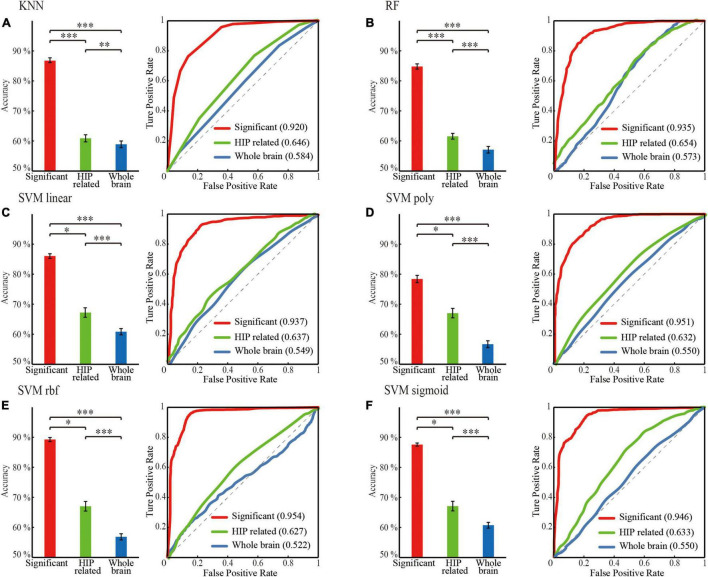
Classification performance comparison between different mean diffusivity (MD) feature sets for different classifier. **(A)** KNN. **(B)** RF. **(C)** SVM linear. **(D)** SVM poly. **(E)** SVM rbf. **(F)** SVM sigmoid. ‘red’: the significant HIP related WM network; ‘green’: HIP related WM network; ‘blue’: the whole brain WM network. Classification performance including mean accuracy (mean ± std; *p < 0.05, **p < 0.005, and ***p < 0.0005; ranksum test with bonferroni correction) and AUC of randomly 100 times hold out method.

**FIGURE 4:**
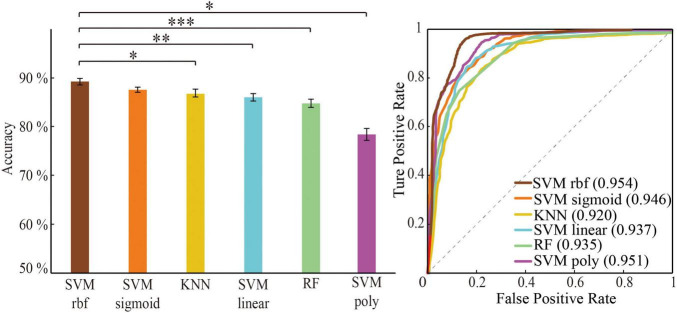
Classification performance comparison between different algorithm through optimal feature set. Classification performance including mean accuracy (mean ± std; *p < 0.05, ^**^p < 0.005, and ^***^p < 0.0005; ranksum test with Bonferroni correction) and AUC of randomly 100 times hold-out method.

**FIGURE 5:**
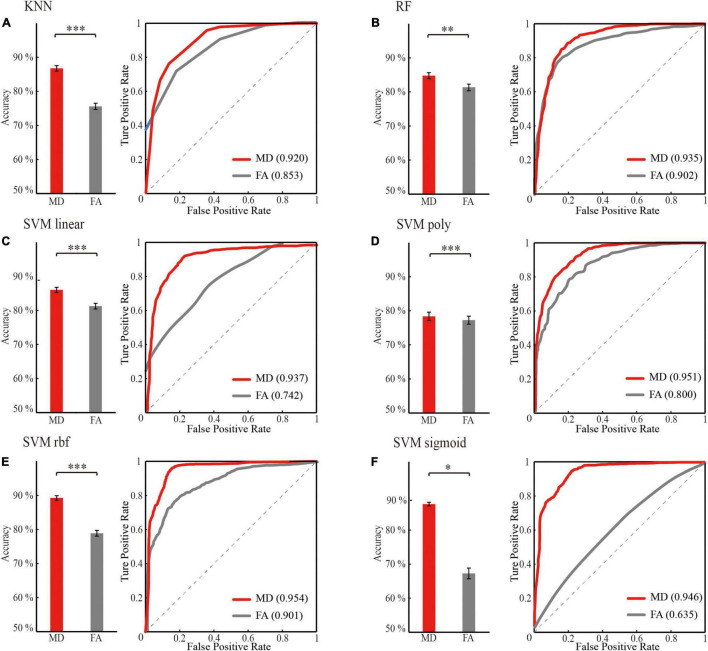
Optimal classification performance comparison between mean diffusivity (MD) and fractional anisotropy (FA) feature sets (the significant HIP related WM network) for different classifier. **(A)** KNN. **(B)** RF. **(C)** SVM linear. **(D)** SVM poly. **(E)** SVM rbf. **(F)** SVM sigmoid. ‘red’: classification performance of mean diffusivity feature sets; ‘gray’: classification performance of fractional anisotropy feature set. Classification performance including mean accuracy (mean ± std; *p < 0.05, **p < 0.005, and ***p < 0.0005; rank sum test with Bonferroni correction) and AUC of randomly 100 times hold out method.

**FIGURE 6:**
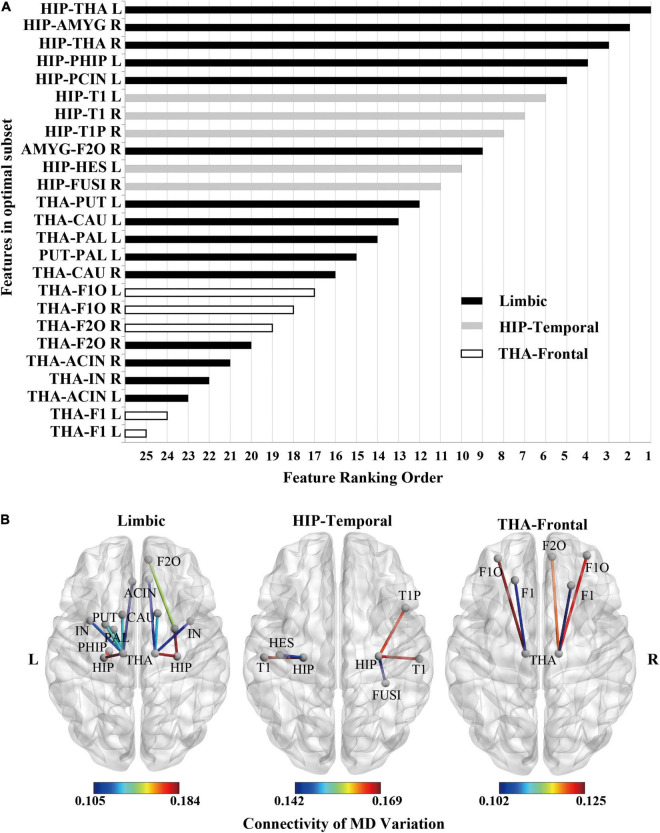
Feature ranking of MD feature (the significant HIP-related WM network) as the result of RFE in SVM rbf model. **(A)** Feature ranking order of the most contributing connectivity (25 features). The bar represents connectivity in different groups. “black”: limbic connectivity (15 features). “gray”: Hip-temporal connectivity (5 features). “white”: THA-frontal connectivity (5 features). **(B)** Schematic illustration of degenerated white matter in MCI. Nodes represented ROIs from AAL templates. Edges represented connectivity; the value represents MD variation, MCI vs. NC.

The following steps were conducted to search for optimal features and classifiers. (1) To demonstrate the effectiveness of the feature extraction method, the classification accuracies (100 times hold-out method) were statistically analyzed in the rank sum test between three features (i.e., whole-brain WM network, HIP-related WM network, and significant HIP-related WM network), and the AUC values were compared in the identical coordinate system (Figure 3). (2) To obtain a better classifier for MCI recognition, the classification accuracies (100 times hold-out method) were statistically analyzed in the rank sum test between different classifiers, and the AUC values were compared in the identical coordinate system (Figure 4). (3) To compare which biomarker is more effective for MCI recognition, the classification accuracies (100 times hold-out method) were statistically analyzed in the rank sum test between MD and FA features, and the AUC values were compared in the identical coordinate system (Figure 5). (4) To rank all features’ contribution to the classification, recursive feature elimination (RFE) was performed. Feature ranking order of the most contributing connectivity was selected based on their classification performance (Figure 6A and Table 4). A schematic illustration of degenerated white matter in MCI was made according to the ranking features (Figure 6B).

## Results

### Participant Characteristics

There were no significant differences in demographic information between the MCI and NC. There were only significant differences in the cognitive scales (CDR and MMSE, Table 1). This indicates that irrelevant variables’ influence was eliminated between the MCI and NC, and the results were credible.

### Regions of Interest Related to Hippocampus in Resting-State Functional Magnetic Resonance Imaging

A total of 43 ROIs related to hippocampus in rs-fMRI were obtained by comparing with AAL (Supplementary Figure 4). Full names and abbreviations of the 43 ROIs were shown in Supplementary Table 1. Among them, 15 ROIs belong to the limbic lobe, 16 ROIs belong to the frontal lobe, 5 ROIs belong to the temporal lobe, 3 ROIs belong to the central region, 3 ROIs belong to the parietal lobe, and 1 ROI belongs to the occipital lobe. Most ROIs are concentrated in the limbic, frontal, and temporal lobes.

### Classification Performance Promoted by Screening Features in Hippocampus-Related Regions of Interest

For the WM network of MD in the same classifier, the performances of progressively optimized features (whole-brain WM network, HIP-related WM network, and significant HIP related WM network) were significantly promoted (*p* < 0.05, rank sum test with Bonferroni correction) (Figure 3 and Table 2). It proved that our feature extraction method in this experiment significantly improved the performance of the MCI classification.

**TABLE 2:** Classification performance based on mean diffusivity (MD) feature sets.

Classifier	Feature	ACC (mean ± sem)	Sen	Spe	AUC
A. KNN	Significant	86.90% ± 0.80%	0.938	0.778	0.920
	HIP related	60.90% ± 1.20%	0.97	0.142	0.646
	Whole brain	58.90% ± 1.10%	0.837	0.265	0.584
B. RF	Significant	84.80% ± 0.90%	0.916	0.781	0.935
	HIP related	61.60% ± 1.00%	0.958	0.144	0.654
	Whole brain	57.10% ± 1.10%	0.935	0.117	0.573
C. SVM linear	Significant	86.20% ± 0.80%	0.905	0.808	0.937
	HIP related	67.30% ± 1.60%	0.886	0.419	0.637
	Whole brain	60.90% ± 1.00%	0.791	0.406	0.549
D. SVM poly	Significant	78.50% ± 1.20%	0.965	0.609	0.951
	HIP related	67.10% ± 1.60%	0.886	0.414	0.632
	Whole brain	56.70% ± 1.20%	0.972	0.029	0.550
E. SVM rbf	Significant	89.40% ± 0.70%	0.938	0.849	0.954
	HIP related	67.10% ± 1.60%	0.887	0.413	0.627
	Whole brain	56.90% ± 1.10%	0.991	0.011	0.522
F. SVM sigmoid	Significant	88.20% ± 0.60%	0.945	0.806	0.946
	HIP related	67.20% ± 1.60%	0.888	0.414	0.633
	Whole brain	60.80% ± 1.00%	0.708	0.501	0.550

*ACC, accuracy; Sen, sensitivity; Spe, specificity; AUC, area under the curve; Ave, average; Std, standard error; Sem, standard error mean.*

### Comparison of Mild Cognitive Impairment Classification Performance Under Different Algorithms

The three machine learning classifiers (of which SVM contains four kernel functions) were compared separately (Figure 4). The performance in order of average classification accuracy (100 times hold-out method) was as follows: SVM rbf (ACC = 89.4%, AUC = 0.954), SVM sigmoid (ACC = 88.2%, AUC = 0.950), KNN (ACC = 86.9%, AUC = 0.920), SVM linear (ACC = 86.2%, AUC = 0.937), RF (ACC = 84.8%, AUC = 0.935), and SVM ploy (ACC = 78.5%, AUC = 0.951). The performance of the SVM rbf was significantly better than other classifiers. Therefore, training with the significant HIP-related WM network as features using SVM rbf can better recognize MCI.

### Comparison of Classification Performance Under Mean Diffusivity and Fractional Anisotropy Features

For the same machine learning classifier, the performance of the feature MD was significantly better than FA (*p* < 0.05, rank sum test with Bonferroni correction) in the case of both using optimal features for training (Figure 5 and Table 3). Therefore, the biomarker MD is superior to FA for MCI diagnosing from a machine learning perspective.

**TABLE 3:** Classification performance of MD and FA feature sets.

Classifier	Feature	ACC (mean ± sem)	Sen	Spe	AUC
A.KNN	MD	86.90% ± 0.80%	0.938	0.778	0.92
	FA	75.70% ± 0.90%	0.905	0.571	0.853
B. RF	MD	84.80% ± 0.90%	0.916	0.781	0.935
	FA	81.40% ± 1.00%	0.878	0.743	0.902
C. SVM linear	MD	86.20% ± 0.80%	0.905	0.808	0.937
	FA	81.40% ± 0.80%	0.891	0.728	0.742
D. SVM poly	MD	78.50% ± 1.20%	0.965	0.609	0.951
	FA	77.50% ± 1.20%	0.877	0.672	0.8
E. SVM rbf	MD	89.40% ± 0.70%	0.938	0.849	0.954
	FA	79.00% ± 0.80%	0.841	0.738	0.901
F. SVM sigmoid	MD	88.20% ± 0.60%	0.945	0.806	0.946
	FA	67.30% ± 1.60%	0.886	0.418	0.635

*ACC, accuracy; Sen, sensitivity; Spe, specificity; AUC, area under the curve; Ave, average; Std, standard error; Sem, standard error mean.*

**TABLE 4:** Feature ranking order for WM connectivity.

Feature ranking order		ROI pairs for connectivity	
1	HIP-THA L	Hippocampus	Thalamus
2	HIP-AMYG R	Hippocampus	Amygdala
3	HIP-THA R	Hippocampus	Thalamus
4	HIP-PHIP L	Hippocampus	Parahippocampal gyrus
5	HIP-PCIN L	Hippocampus	Posterior cingulate
6	HIP-T1 L	Hippocampus	Superior temporal gyrus
7	HIP-T1 R	Hippocampus	Superior temporal gyrus
8	HIP-T1P R	Hippocampus	Temporal pole: superior temporal gyrus
9	AMYG-F2O R	Amygdala	Middle frontal gyrus, orbital part
10	HIP-HES L	Hippocampus	Heschl gyrus
11	HIP-FUSI R	Hippocampus	Fusiform gyrus
12	THA-PUT L	Thalamus	Lenticular nucleus, putamen
13	THA-CAU L	Thalamus	Caudate nucleus
14	THA-PAL L	Thalamus	Lenticular nucleus, pallidum
15	PUT-PAL L	Lenticular nucleus, putamen	Lenticular nucleus, pallidum
16	THA-CAU R	Thalamus	Caudate nucleus
17	THA-F1O L	Thalamus	Superior frontal gyrus, orbital part
18	THA-F1O R	Thalamus	Superior frontal gyrus, orbital part
19	THA-F2O R	Thalamus	Middle frontal gyrus, orbital part
20	THA-IN L	Thalamus	Insula
21	THA-ACIN R	Thalamus	Anterior cingulate and paracingulate gyri
22	THA-IN R	Thalamus	Insula
23	THA-ACIN L	Thalamus	Anterior cingulate and paracingulate gyri
24	THA-F1 L	Thalamus	Superior frontal gyrus, dorsolateral
25	THA-F1 R	Thalamus	Superior frontal gyrus, dorsolateral

### Ranking the Contribution of White Matter Structural Connectivity to Mild Cognitive Impairment Classification

According to the significant HIP-related WM network’s feature ranking order through RFE (Figure 6A and Table 4), schematic illustrations of degenerated white matter in MCI were made (Figure 6B). The most common connections between MCI and NC are HIP-temporal connectivity, limbic connectivity, and THA-frontal connectivity.

## Discussion

This study proposes a feature extraction method for whole-brain white matter connectivity networks driven by ROIs related to the hippocampus. The whole-brain WM network, HIP-related WM network, and significant HIP-related WM network were obtained in the process of feature extraction and optimization. Different classification algorithms, such as KNN, RF, and SVM (linear, poly, rbf, sigmoid), were used to test the classification performance. The pathological mechanisms of MCI were also revealed by RFE. Our study found that feature extraction of whole-brain white matter connectivity by hippocampus-related brain regions can significantly improve MCI classification performance. It can be summarized in three points:

1. In the feature, compared with the whole-brain WM network, the HIP-related WM network can significantly promote the MCI classification performance in machine learning.
2. In the algorithm, the classification performance of the SVM rbf is optimum when taking significant HIP-related WM networks as features.
3. In the pathology, the hippocampus- and thalamus-related white matter connectivity greatly contributed to MCI recognition. So, the method that combines MCI pathology and uses a suitable classifier can significantly improve the classification performance, while the ranking of features contribution can reveal MCI pathology in turn.

### Mild Cognitive Impairment Classification Performance Significantly Promoted by Feature Extraction of Hippocampus-Related Regions of Interest

For the same classifier on features, the performance on MCI classification was significantly higher for features screening by HIP-related ROIs than the whole brain. First, in our results, the whole-brain WM network, HIP-related WM network, and significant HIP-related WM network had sequentially significantly higher classification performance (Figure 3). Second, in terms of AD pathogenesis, the degeneration of MCI first appears in specific fiber tracts and gradually spreads to the whole brain when developing to the AD stage (Daianu et al., 2015; Jones et al., 2015; Wang et al., 2016). Furthermore, in either specific fiber tracking (Nir et al., 2015; Dou et al., 2020) or whole-brain white matter connectivity network measures (Wee et al., 2011; Prasad et al., 2015) in previous studies, the accuracies of MCI classification were around 60%, while the accuracies of AD can be about 80%. It also corroborates the pathogenesis of AD from an engineering perspective. Finally, for MCI recognition, a single use of whole-brain white matter would weaken the features considering AD’s pathogenesis. Based on the pathology of MCI, our study improves the MCI classification performance effectively by feature extraction of HIP-related ROIs.

### Mean Diffusivity Can Be a Valid Biomarker for Mild Cognitive Impairment Recognition

For MCI, the MD is more sensitive than FA values to reflect white matter degeneration. First, our results showed that the MD of WM networks as features outperformed FA in all the machine learning classifiers (Figure 5). Second, research showed the sensitivity of the MD index to MCI (Yu et al., 2017), while FA only begins to have statistical differences in multiple fiber tracts during AD (Mito et al., 2018). Our experiment confirmed that the MD index was more sensitive in the MCI stage from classification performance. Furthermore, FA reflects the density of white matter fibers and is influenced by the axon diameter of fiber tracts. MD represents the integrity of the myelin outer the fiber tracts. Demyelination can cause MD to increase (Tournier, 2019). It can be inferred that disintegrated myelin appeared more obvious than axon loss during MCI. Therefore, the MD index can be an effective biomarker for MCI compared with FA.

### Comparison With Previous Studies

Compared with previous studies that used white matter as the feature to recognize MCI, our method using HIP-related ROIs to extract white matter features significantly improved MCI classification performance (Table 5). By comparing the classification performance between all of our classifiers, the SVM classifier with rbf kernel significantly outperformed the other classifiers (Figure 4). It can also be seen from previous studies that the SVM classifier with rbf kernel for MCI recognition performed well when taking the white matter as features (Table 5). Therefore, training with the white matter as features after HIP-related ROIs extraction can better recognize MCI when using the SVM rbf classifier.

**TABLE 5:** Summary of the studies using dMRI features for MCI classification.

Comparison with the previous studies	Classifier	Subjects	Feature	Database	Performance	
		MCI/NC			ACC	AUC
Ebadi et al. (2017)	KNN	15/15	Network	Local	60.0%	0.560
Our study	KNN	42/54	Network	Local	86.9%	0.920
Maggipinto et al. (2017)	RF	90/89	MD/FA voxel	ADNI	54.0%	0.600
Wang et al. (2018)	RF	169/379	Network	ADNI/NACC	75.0%	0.850
Our study	RF	42/54	Network	Local	84.8%	0.935
Xie et al. (2015)	SVM linear	64/64	MD/FA voxel	Local	78.9%	0.856
Our study	SVM linear	42/54	Network	Local	86.2%	0.937
Cui et al. (2012)	SVM rbf	79/204	FA voxel	SMA	71.1%	0.700
Dyrba et al. (2015a)	SVM rbf	35/42	MD/FA voxel	EDSD	77.0%	0.680
Demirhan et al. (2015)	SVM rbf	43/70	FA voxel	ADNI	78.5%	0.758
Nir et al. (2015)	SVM rbf	113/50	Network	ADNI	79.0%	-
Ahmed et al. (2017)	SVM rbf	58/52	MD voxel	ADNI	79.4%	0.788
Our study	SVM rbf	42/54	Network	Local	89.4%	0.954

*local, collect by hospital; ADNI, Alzheimer’s Disease Neuroimaging Initiative; NACC, National Alzheimer’s Coordinating Center; SMA, Sydney Memory and Aging; EDSD, European DTI Study on Dementia; -, not applicable.*

### Important Hubs of the White Matter Connectivity Network for Mild Cognitive Impairment: Hippocampus and Thalamus

White matter connections related to hippocampal and thalamic contribute most to MCI classification. First, previous studies have shown that the white matter associated with the hippocampus and thalamus degenerates during MCI. The white matter microstructure between the hippocampus and medial temporal lobe changes during MCI (Teipel et al., 2016; Zhuo et al., 2016; Dumont et al., 2019). The fornix connects the hippocampus to the thalamus, and its white matter degeneration leads to decreased memory function (Christiansen et al., 2016). Degeneration of the projection tracts connecting the thalamus to the prefrontal lobes leads to a decrease in executive function (Gu and Zhang, 2019; Liu et al., 2021).

Second, in our results, features connected with the hippocampus or thalamus in the white matter connectivity network ranked high in contributions for MCI classification, and features connected with the hippocampus ranked more advanced than the thalamus (Figure 6). This result also confirmed our previous findings (Zhou et al., 2021). In our previous study, the MD of all voxels in a single fiber tract was taken as features. The fiber tract with high separability for MCI recognition passed through the hippocampus or thalamus. So, whether single fiber tract or whole-brain network had corroborated that hippocampus and thalamus were important hubs of the white matter connectivity network for MCI.

Furthermore, in our previous study, the highest average accuracy for MCI classification reached 71.0% when taking MD of all voxels in the left inferior longitudinal fasciculus. In this study, the highest average accuracy reached 89.4% by taking white matter networks related to the hippocampus. Compared with the single fiber tract, white matter networks related to the hippocampus as features can improve classification performance. Finally, the contribution of every white matter connectivity can be sorted by RFE. Thus, the hippocampus and thalamus are important hubs for MCI. Features of combinational white matter connectivity networks outperform single fiber tracts.

### Limitations

This study mainly focused on feature extraction for MCI recognition. However, the valuable contributions of this study must be considered within the context of certain limitations. First, a certain amount of new data will be added as the test set alone. Data in this article will be used as the training set and validation set. Second, cross-validation will be added to obtain more accurate parameters for the classifier further to improve performance in the future. Third, deep learning will be used based on our existing findings, and generalization could be improved in the future. More subjects and multiple datasets will be acquired from different hospitals to test the generalization of the classifier. Furthermore, future studies should recruit participants with both MCI and AD. The NC, MCI, and AD should be divided to investigate the pathological mechanisms underlying AD development. Finally, the behavior scale would be added, and the support vector regression (SVR) will be used to predict MCI patients’ behavior.

## Conclusion

Our study proposes a feature extraction method driven by hippocampus-related ROIs for white matter connectivity networks. In the feature extraction process, the whole-brain WM network, the HIP-related network, and the significant HIP-related network continuously optimize the performance of MCI classification. By recursive feature elimination, the pathological mechanism revealed that the hippocampus and thalamus are important hubs in white matter networks for MCI. Our results provide a valid basis for the early diagnosis of AD.

## Data Availability Statement

The original contributions presented in the study are included in the article/Supplementary Material. The main codes that support the findings of this study are available on GitHub (https://github.com/ThreePoundUniverse/MCI-network-classification). Further inquiries can be directed to the corresponding authors.

## Ethics Statement

The studies involving human participants were reviewed and approved by Institutional Review Board of Tianjin University; Ethics Committee of Chang Gung University. The patients/participants provided their written informed consent to participate in this study.

## Author Contributions

XS and YZ designed and conceptualized the research and wrote the manuscript. YZ, Y-PC, and C-PL collected the data. YZ, YC, SL, XZ, YS, and XS analyzed the data. XS, DM, and QL supervised the study. All authors contributed to the article and approved the submitted version.

## Conflict of Interest

The authors declare that the research was conducted in the absence of any commercial or financial relationships that could be construed as a potential conflict of interest.

## Publisher’s Note

All claims expressed in this article are solely those of the authors and do not necessarily represent those of their affiliated organizations, or those of the publisher, the editors and the reviewers. Any product that may be evaluated in this article, or claim that may be made by its manufacturer, is not guaranteed or endorsed by the publisher.

## Footnotes

1
http://www.brain.org.au/software/mrtrix/

2
https://fsl.fmrib.ox.ac.uk/fsl

3
http://www.fil.ion.ucl.ac.uk/spm

4
http://restfmri.net/forum/REST

5
http://dsi-studio.labsolver.org/

6
https://scikit-learn.org/
