# Supplementary material for: Automated Classification of Mild Cognitive Impairment by Machine Learning With Hippocampus-Related White Matter Network
Source: Front Aging Neurosci. 2022 Jun 14;14:866230. doi: 10.3389/fnagi.2022.866230 (PMC9237212; doi:10.3389/fnagi.2022.866230)
Supplement: Supplementary file 1 [file Data_Sheet_1.pdf]

Supplementary Materials

Supplementary information	The details in Materials
Supplementary Figure S1	Establishment for whole brain WM network.
Supplementary Figure S2	Transformation matrix between native and standard space.
Supplementary Figure S3	Selection of hippocampus related ROI.
Supplementary Figure S4	ROIs related to hippocampus.
Supplementary Table S1	Hippocampus related ROIs

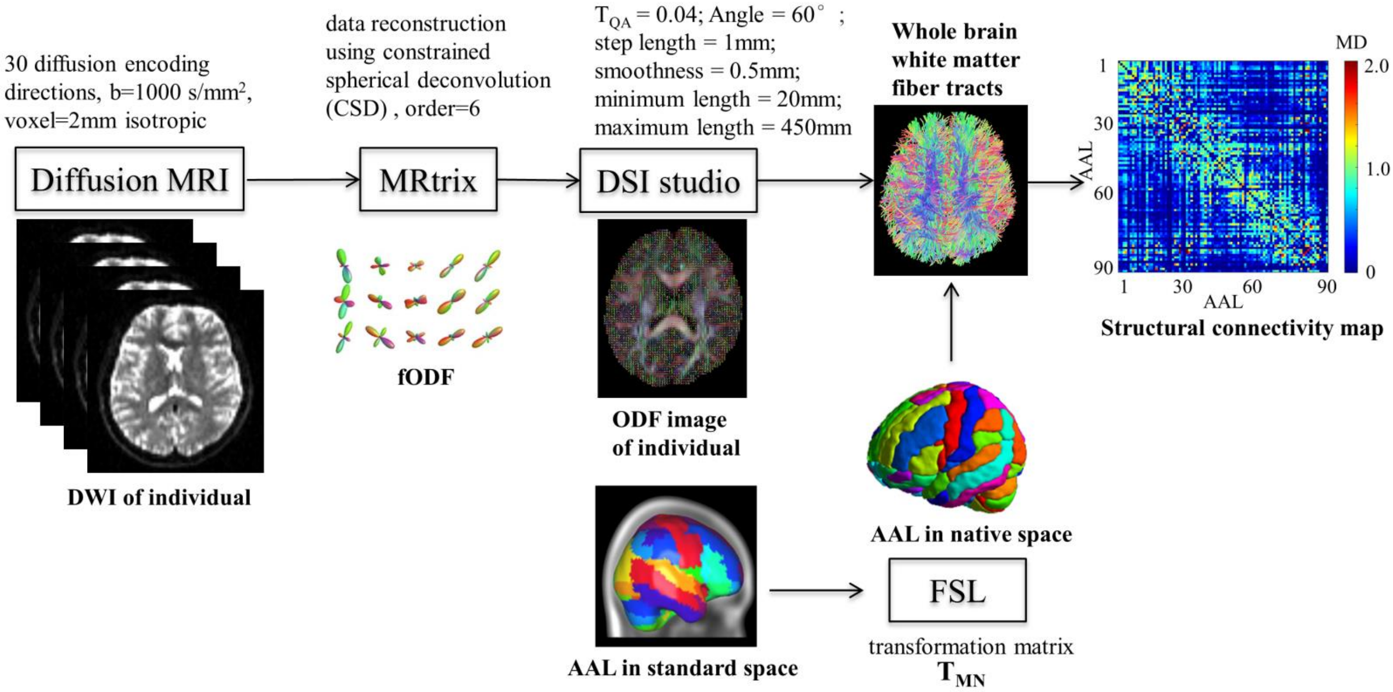

Figure S1. Establishment for whole brain WM network. The whole brain white matter fiber tracts were tracked by DTI with 30 diffusion encoding directions. The whole brain WM network was calculated between the AAL template.

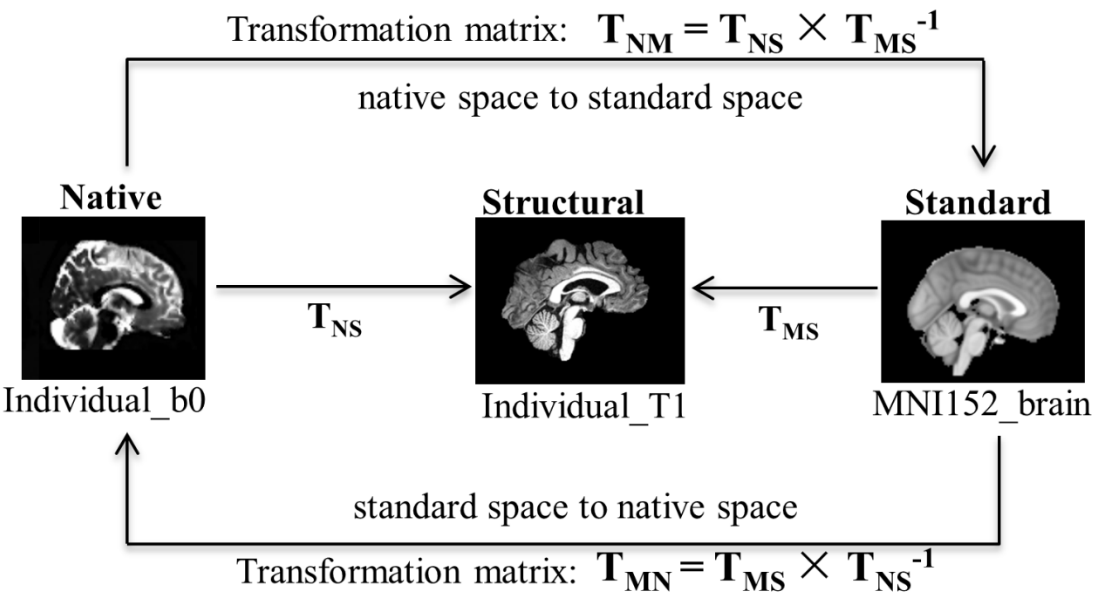

Figure S2. Transformation matrix between native and standard space. The transformation matrix that native space to standard space is T_{NM}. Its inverse matrix that standard space to native space is T_{MN}.

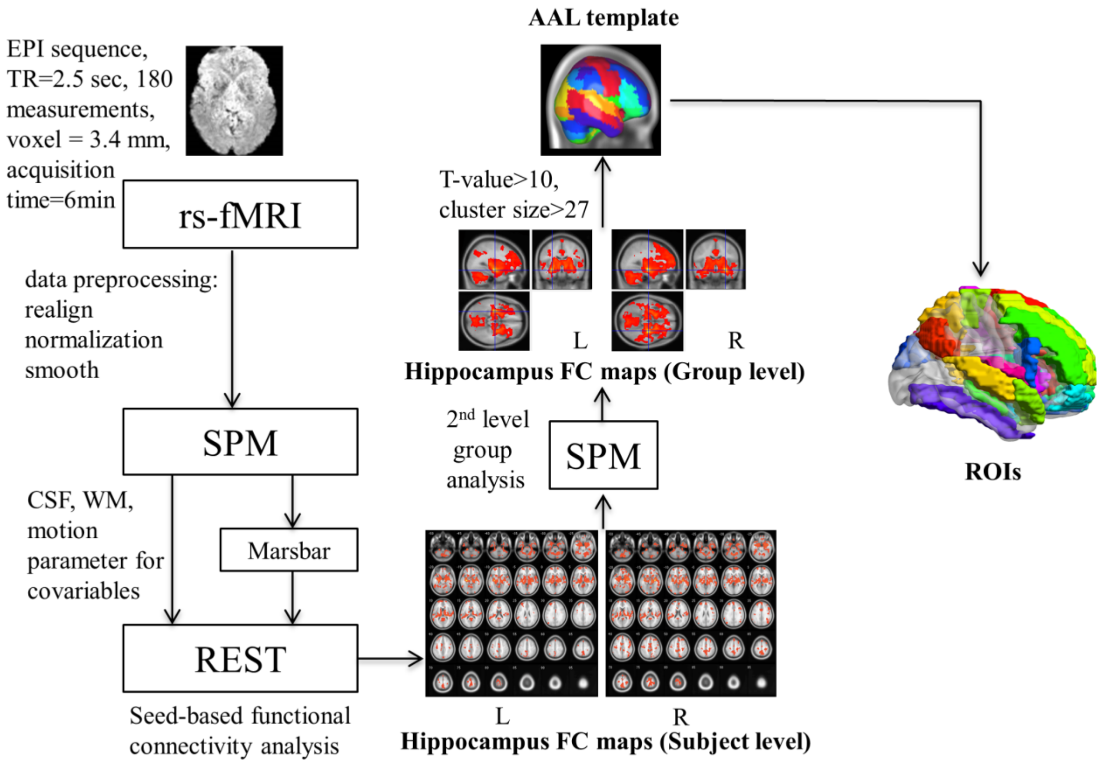

Figure S3. Selection of hippocampus related ROI. The hippocampus served as the seed points to create the FC maps and then compared with the AAL template to select ROIs.

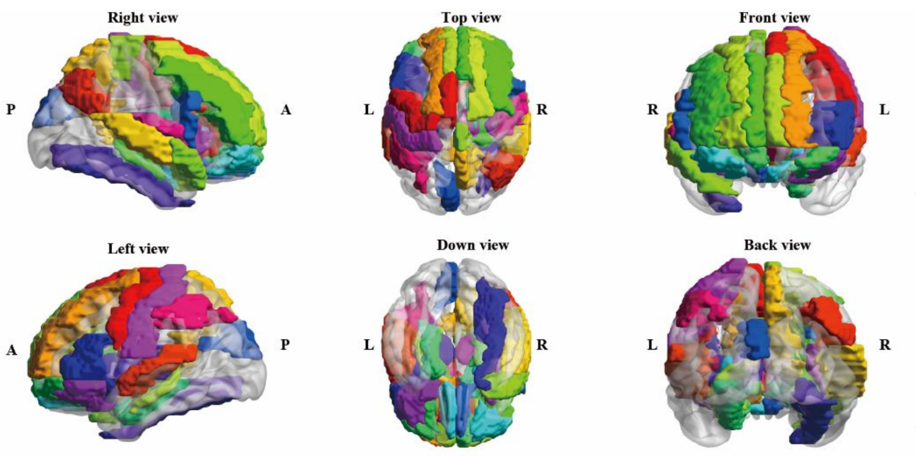

Figure S4. ROIs related to hippocampus. 15 ROIs in Limbic lobe; 16 ROIs in Frontal lobe; 5 ROIs in Temporal lobe; 3 ROIs in Central region; 3 ROIs in Parietal lobe; 1 ROIs in Occipital lobe.(**Table S1**)

Table S1. Hippocampus related ROIs

Lobe	Full name	Abbr	AAL label
Limbic (15 ROIs)	Insula (L/R)	IN	29/30
	Anterior cingulate and paracingulate gyri (L/R)	ACIN	31/32
	Posterior cingulate gyrus (L)	PCIN	35
	Hippocampus (L/R)	HIP	37/38
	Parahippocampal gyrus (L)	PHIP	39
	Amygdala (R)	AMYG	42
	Caudate nucleus (L/R)	CAU	71/72
	Lenticular nucleus, putamen (L)	PUT	73
	Lenticular nucleus, pallidum (L)	PAL	75
	Thalamus (L/R)	THA	77/78
Frontal (16 ROIs)	Superior frontal gyrus, dorsolateral (L/R)	F1	3/4
	Middle frontal gyrus (R)	F2	8
	Superior frontal gyrus, orbital part (L/R)	F1O	9/10
	Inferior frontal gyrus, opercular part (R)	F3OP	12
	Inferior frontal gyrus, triangular part (L)	F3T	13
	Inferior frontal gyrus, orbital part (L)	F3O	15
	Supramarginal gyrus (L)	SMA	19
	Olfactory cortex (L)	OC	21
	Superior frontal gyrus, medial (L/R)	F1M	23/24
	Middle frontal gyrus, orbital part (R)	F2O	26
	Gyrus rectus (L/R)	GR	27/28
	Paracentral lobule (R)	PCL	70
Temporal (5 ROIs)	Fusiform gyrus (R)	FUSI	56
	Heschl gyrus (L)	HES	79
	Superior temporal gyrus (L/R)	T1	81/82
	Temporal pole: superior temporal gyrus (R)	T1P	84
Central (3 ROIs)	Precentral gyrus (L)	PRE	1
	Rolandic operculum (R)	RO	18
	Postcentral gyrus (L)	POST	57
Parietal (3 ROIs)	Inferior parietal, but supramarginal and angular gyri (L)	P2	61
	Angular gyrus (R)	AG	66
	Precuneus (R)	PQ	68
Occipital (1 ROIs)	Cuneus (L)	Q	45

Note: Abbr: abbreviation of the ROI's in AAL; AAL label: number of the ROI in automated anatomical labeling; odd number: ROI in the left hemisphere; even number: ROI in the right hemisphere.
